# The Supera Interwoven Nitinol Stent as a Flow Diverting Device in Popliteal Aneurysms

**DOI:** 10.1007/s00270-022-03118-x

**Published:** 2022-04-04

**Authors:** L. van de Velde, E. Groot Jebbink, B. A. Zambrano, M. Versluis, J. Tessarek, M. M. P. J. Reijnen

**Affiliations:** 1 Department of Vascular Surgery, Rijnstate Hospital, Arnhem, The Netherlands; 2 Physics of Fluids Group, TechMed Centre, University of Twente, Drienerlolaan 5, 7522 NB Enschede, The Netherlands; 3 M3i Multi-Modality Medical Imaging Group, TechMed Centre, University of Twente, Enschede, The Netherlands; 4 J. Mike Walker ’66 Department of Mechanical Engineering, Texas A&M University, College Station, TX USA; 5 Department of Vascular Surgery, Bonifatius Hospital, Lingen, Germany

**Keywords:** Popliteal artery, Self-expandable metallic stents, Aneurysm, Platelet activation

## Abstract

**Purpose:**

The feasibility of using a compressed interwoven Supera stent as a flow diverting device for popliteal aneurysms was recently demonstrated in patients. It is unclear, however, what the optimal flow diverting strategy is, because of the fusiform shape of popliteal aneurysms and their exposure to triphasic flow. To assess this flow diverting strategy for popliteal aneurysms, flow profiles and thrombus formation likelihood were investigated in popliteal aneurysm models.

**Materials and Methods:**

Six popliteal aneurysm models were created and integrated into a pulsatile flow set-up. These models covered a bent and a straight anatomy in three configurations: control, single-lined and dual-lined Supera stents. Two-dimensional flow velocities were visualized by laser particle image velocimetry. In addition, the efficacy of the stent configurations for promoting aneurysm thrombosis was assessed by simulations of residence time and platelet activation.

**Results:**

On average for the two anatomies, the Supera stent led to a twofold reduction of velocities in the aneurysm for single-lined stents, and a fourfold reduction for dual-lined stents. Forward flow was optimally diverted, whereas backward flow was generally deflected into the aneurysm. The dual-lined configuration led to residence times of 15–20 s, compared to 5–15 s for the single stent configurations. Platelet activation potential was not increased by the flow diverting stents.

**Conclusion:**

A compressed Supera stent was successfully able to divert flow in a popliteal aneurysm phantom. A dual-lined configuration demonstrated superior hemodynamic characteristics compared to its single-lined counterpart.

**Supplementary Information:**

The online version contains supplementary material available at 10.1007/s00270-022-03118-x.

## Introduction

Endovascular repair with a covered stent is an established treatment for popliteal artery aneurysms (PAA), with a 5-year primary patency rate of 60–69% and a secondary patency rate of 71–77% [1, 2]. A disadvantage of this approach is the difficulty of bridging a large diameter mismatch between landing zones, as well as overstenting sidebranches, in particular the anterior tibial artery. In a prospective cohort, about 10% of PAA’s were excluded from treatment with a covered stent due to an inadequate landing zone [3]. Furthermore, stent fractures occur with a reported incidence of 19% at a median follow-up of 40 months, associated with clinical symptoms in 38% of cases [2].

**Fig. 1:**
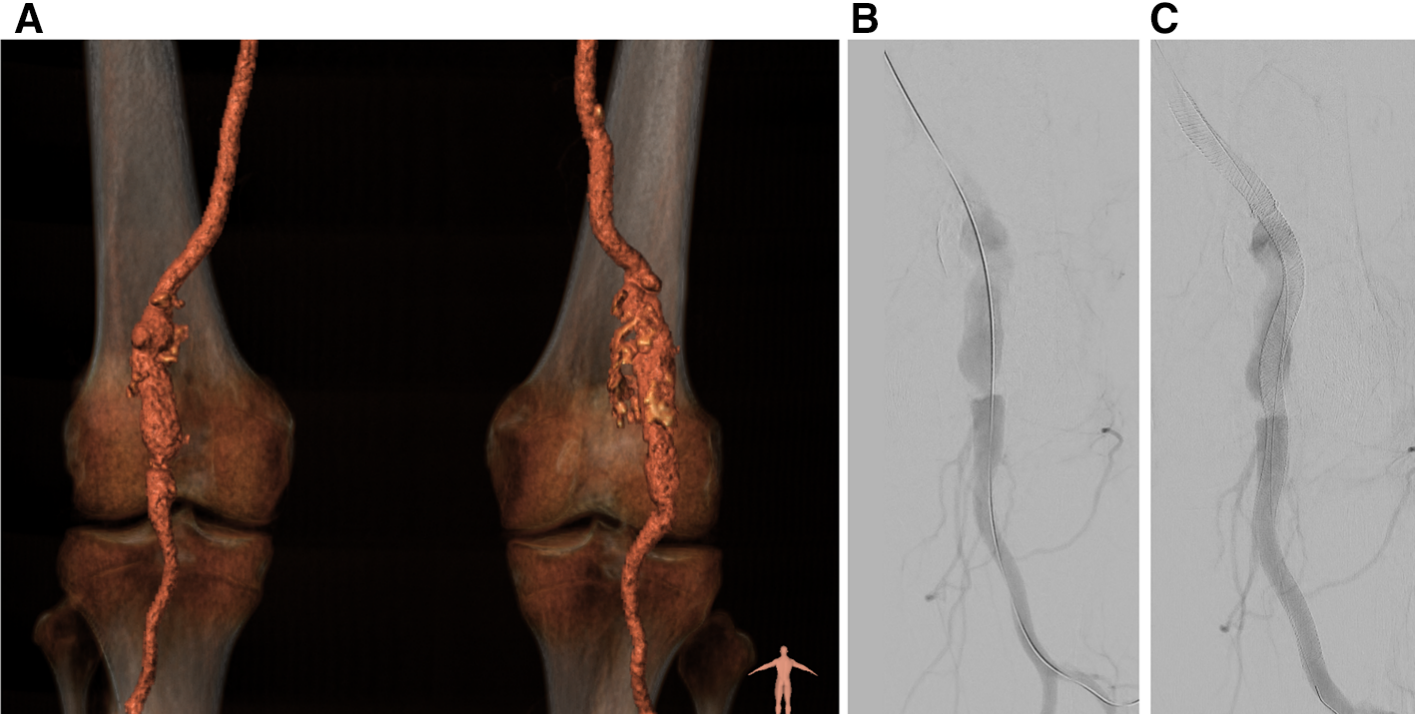
Clinical case example of a treated popliteal aneurysm. **A** posterior view of CTA showing bilateral popliteal aneurysm with curved inlet. **B** and **C** DSA of right popliteal aneurysm before (**B**) and after (**C**) treatment. Video 1 shows the telescoping deployment of the Supera stent

An alternative strategy is the use of a flow diverting stent, i.e. a bare stent with high strut density. In cerebral aneurysms, flow diverters effectively reduce flow in the aneurysm and promote aneurysm thrombosis and subsequent shrinkage [4]. PAA can be treated in this fashion using the Supera interwoven nitinol stent [5] (Abbott, Santa Clara, CA). During deployment, the stent can be locally compressed to achieve a high mesh density (Fig. 1; Video 1), creating a flow diverting configuration that can be augmented by using multiple Supera stents. In two PAA cohorts treated with 1–3 Supera stent layers, the 1-year patency was 85–100% and at median 44-month follow-up, patency was 67% [5, 6]. Aneurysm thrombosis was seen in 88% within a week, and in 100% through 4-month follow-up [5]. Potential advantages include increased flexibility with respect to diameter transitions and patency of overstented side-branches.

How the Supera flow diverting configuration alters hemodynamics in a PAA has not yet been investigated. Flow diverters are expected to decrease velocities in the aneurysm, but it is unknown whether this holds for PAA, where flow reversal, i.e. a diastolic retrograde flow, is typically present. Aneurysm thrombosis is a biological process to which both high shear rates and stasis of blood may contribute. Flow diversion may stimulate both routes, by augmenting particle residence time in the aneurysm, or by shear-induced platelet activation when blood is expulsed through the stent struts [7]. An in-depth understanding of how blood flow is altered by different Supera stent configurations would help optimize its clinical application. To assess in which configuration a flow-diverting Supera stent optimally promotes flow conditions that induce thrombosis, this study explored how a single and dual-lined Supera stent altered flow characteristics, residence time and platelet activation in PAA phantoms.

## Materials and Methods

### In-Vitro Model Construction

**Fig. 2:**
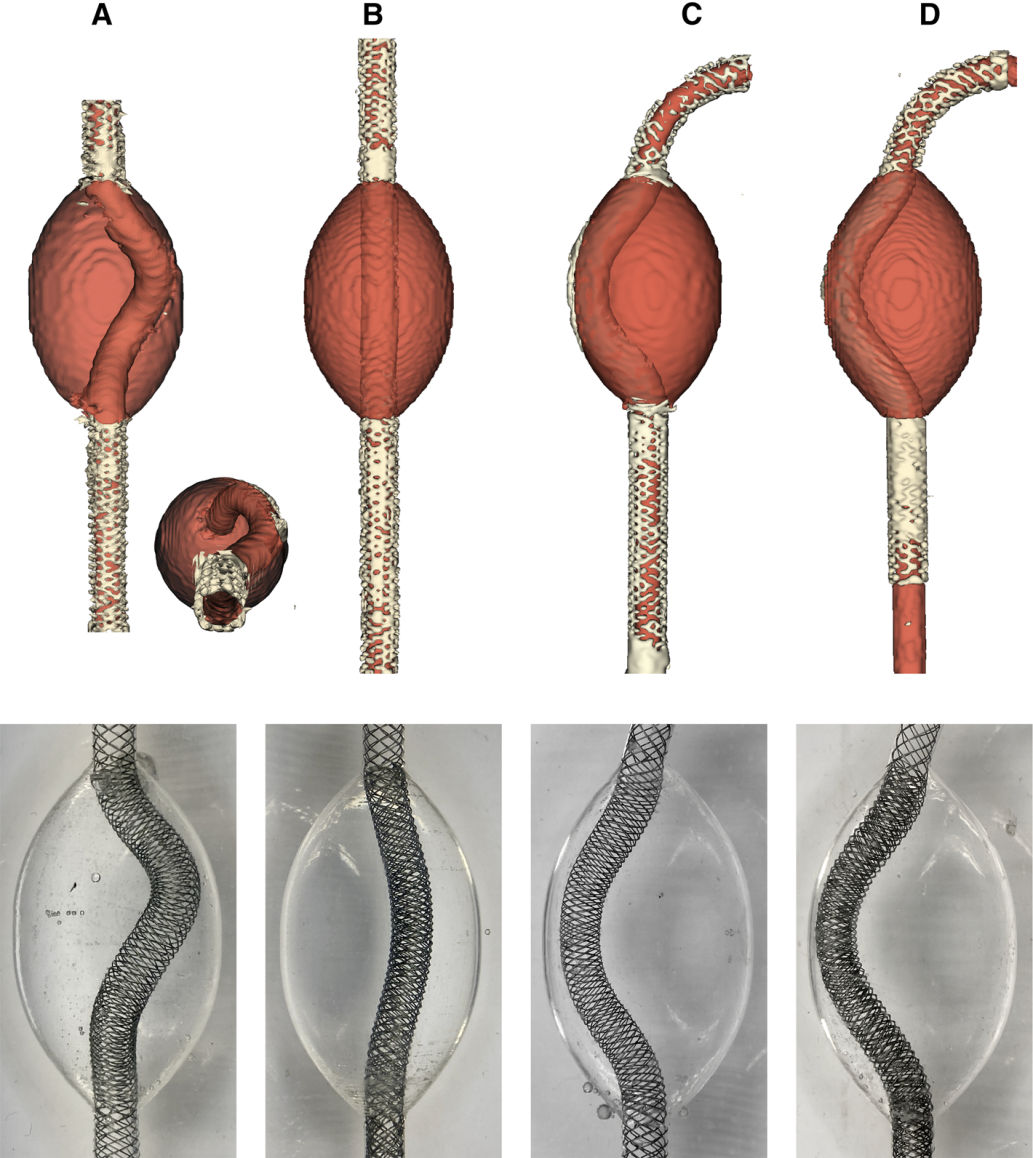
Segmentations (top) and photographs (bottom) of the four stented models. **A** straight inlet with single Supera stent; the top-down barrel view in the right-down insert shows the tortuous stent trajectory, **B** straight inlet with dual-lined Supera stent, **C** bent inlet with single Supera stent, **D** bent inlet with dual-lined Supera stent. Video 2 shows the deployment of the Supera stent in the phantom

Six rigid PAA models were created and integrated into a flow set-up that reproduced physiologic pulsatile flow. The anatomy and flow volumes were based on a cohort of 10 patients with an indication for PAA exclusion. Patient-averaged anatomies yielded a 30-mm aneurysm with a 50-mm length. Two anatomies were designed (Fig. 2), corresponding to a PAA for a knee in an extended and bent position [8].

For both anatomies, three stent configurations (no stent, single lining and dual aneurysm lining) were established. Single stent deployment with maximal strut compression in the aneurysm resulted in a helical stent trajectory and a simpler curved stent trajectory in the straight and bent anatomy, respectively (Fig. 2, Video 2). The dual stent deployment led to a straightened stent trajectory in the straight anatomy, whereas the curved trajectory in the bent anatomy was unaltered.

**Fig. 3:**
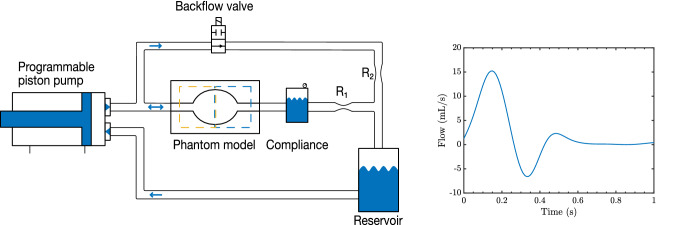
Physiologic flow loop (left) used to recreate pulsatile triphasic flow (right) as present in the popliteal aneurysm cohort. Flow is pushed forward into the model by the piston pump, pressurizing the air in the compliance chamber, aided by the high resistance R_1_. At the end of systole, the solenoid backflow valve opens, which creates a low-resistance pathway. The flow through the phantom model now reverses, as the liquid moves from the high-pressure compliance through the phantom model, backflow valve and low R_2_-resistance into the reservoir

The model was placed in a circulating flow set-up with a blood-mimicking fluid (Fig. 3). At the inlet, a hydraulic piston pump (SuperPump, ViVitro labs, Victoria, Canada) was used to enforce time-varying forward flow. An electronically controlled valve along with a Windkessel was used to reproduce triphasic flow derived from duplex-ultrasound. The amplitude of the pump was set such that a flow volume of 2.9 mL was ejected during forward flow, of which 0.7 mL returned during the backflow phase. The supplementary material provides more details on the anatomy and flow profiles from the selected patient cohort, as well as on the flow set-up.

### Particle Image Velocimetry

Fluorescent particles were added to the blood-mimicking fluid [9] and illuminated by a 1-mm laser sheet that captured the stent trajectory as much as possible. Two adjacent camera windows of 30 × 30 mm (Fig. 3) were recorded, capturing the full aneurysm and the landing zones. To derive 2D velocity vector fields with 0.23 mm interspacing, particle image velocimetry analysis was performed with PIVlab [10] with a three-pass cross-correlation analysis [9]. For both camera windows, flow was recorded for 10 cycles and ensemble-averaged to produce a velocity field for one cardiac cycle, which additionally filtered cyclic variations in jet breakdown. Subsequently, vectors from the two camera windows were merged. A local median filter was applied to vectors on the intersection of the imaging windows, which discarded 5–15% of the vectors, demonstrating acceptable agreement. From the velocity field, time-averaged wall shear stress (WSS) was computed [9].

### Thrombosis Prediction

To quantify the likelihood of thrombosis, particle pathlines were computed from a cycle-averaged velocity field. Two simulations were performed to analyze different thrombotic initiators: platelet activation and residence time of coagulation factors.

For the residence time simulation, particles were uniformly seeded throughout the model and tracked for 20 cycles. Residence time (RT) was computed for 1 × 1 mm^2^ sections of the aneurysm, defined as the time elapsed for the particle concentration to fall under 2% of the initial seeding concentration [11].

To assess platelet activation, particles were seeded near the wall and tracked backwards in time for a maximum of 20 cycles [12], whilst computing the shear modulus that a particle experienced per timepoint to evaluate the platelet activation potential (PLAP) [13].

Platelet activation depends on the combination of shear stress and exposure time, with both short-lasting high shear stress and long-lasting medium shear stress able to activate platelets. At the maximum shear stress levels of ~ 200 Pa in the model, the activation threshold for the product of shear stress and exposure time is in the order of 20 Pa.s [14]. The supplementary material provides more details on platelet and residence time simulations.

## Results

**Fig. 4:**
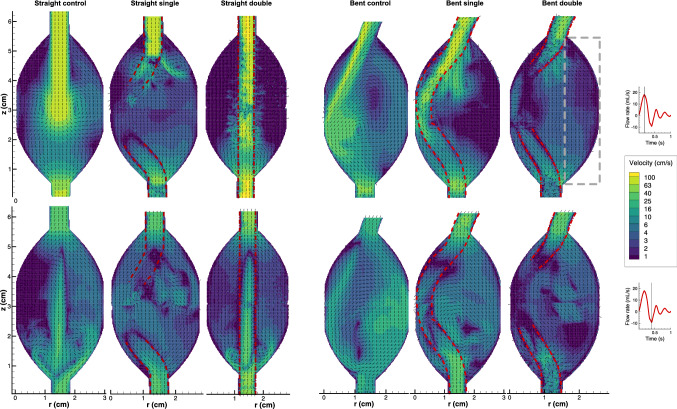
Velocity vector and contour plots of the popliteal models. Top: peak systole; bottom: backflow. The single-lined Supera stent is out-of-plane halfway in the aneurysm. The color scale corresponds to a logarithmic distribution of the velocity magnitude. The flow rate (right) was calculated from the vector field in the inlet of the control model, assuming axisymmetric flow. In the bent dual model (upper right), the grey rectangle demarcates the area from which aneurysm velocities were extracted. Video 3A and 3B show animated flow velocity vectors for the full cycle

The velocity vector fields during peak forward and peak backward flow are plotted in Fig. 4. Time evolution of the velocity field is provided in video 3A (straight) and 3B (bent). For all models, velocities and WSS were extracted from a window covering the right half of the unstented aneurysm (Fig. 4). Time-averaged statistics for the extracted velocities and WSS are summarized in Table 1.

**Table 1:** Velocity and wall shear stress distribution in the right half of the aneurysm

	Velocity magnitude (cm/s)		Time-averaged wall shear stress (Pa)
	Median	Interquartile range	Mean
Straight control	2.5	1.4–3.6	0.31
Straight single-lined	1.3	0.7–2.1	0.12
Straight dual-lined	0.8	0.4–1.4	0.12
Bent control	5.8	3.7–8.2	0.19
Bent single-lined	1.5	0.8–2.9	0.17
Bent dual-lined	1.1	0.6–2.0	0.04

### Control Models

For the straight control model, an eminent systolic jet produced modest vortices which induced flow in the aneurysm (Fig. 4). Upon maximal backward flow, the systolic jet remnants still forced flow forward in the center, while backward flow was routed through the sides of the aneurysm. In the bent control model, the systolic jet entered the aneurysm at an angle and impinged the aneurysm wall (Fig. 4). A prominent counterclockwise vortex was continuously present in this model (Video 3B). During flow reversal, backflow traversed through the right half of the aneurysm.

### Stented Models

In the straight model, the helical trajectory of the single-lined Supera stent (Fig. 2) led to out-of-plane flows with a less organized flow (Video 3A) compared to the control model. Nonetheless, velocities were reduced by a factor 2 relative to the control model (Table 1). In the dual-lined straight model, flow velocity was reduced by an additional 38%. In this model, especially systolic flow velocities were reduced (Fig. 4), with only small puffs moving laterally outward from interstrut spaces. During flow reversal, the systolic jet’s momentum retained forward flow in the stent, forcing backflow to move through the full width of the aneurysm. Compared to the single-lined stent, significantly weaker jets (10 vs 30 cm/s) were pushed laterally outward from the stent struts during systole, a trend that was reversed during backflow (25 vs 10 cm/s).

In the bent model, the single Supera stent pierced the incoming systolic jet (Fig. 4), generating small jets that protruded from the stent into the aneurysm. During flow reversal, the backward flow mixed with the vorticity generated during systole, creating complex flow features (Video 3B). For the dual-lined bent model, similar observations held, albeit with less strong jets emanating through the stent during systole (Fig. 4). The single and dual Supera stent resulted in a fourfold and fivefold reduction in median velocity, respectively (Table 1).

In the straight models, both a single and dual Supera stent led to a 61% decrease in WSS. For the bent models, a single and dual Supera stent caused an 11% and a 79% reduction in WSS, respectively.

### Thrombosis Potential

**Fig. 5:**
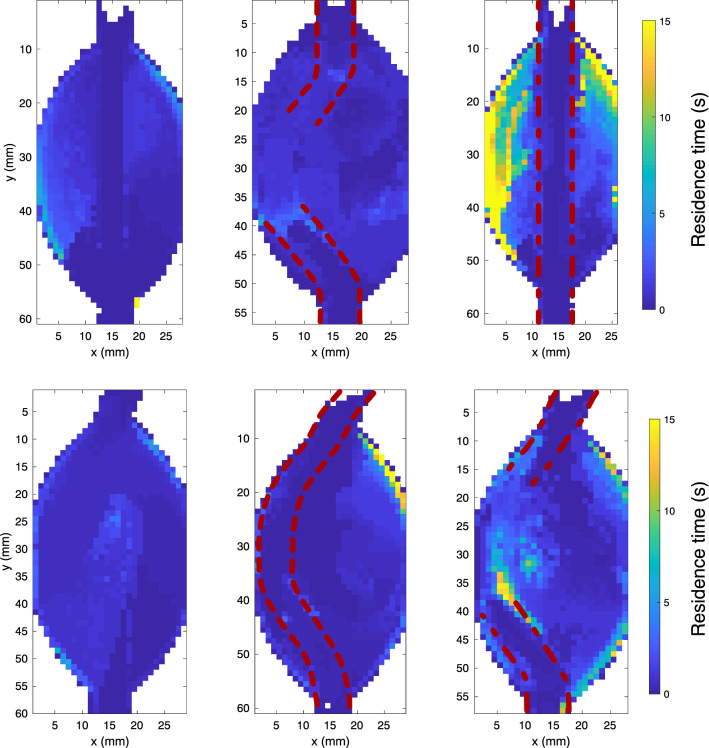
Local residence time based on computed particle pathlines in the models. Top: straight models, bottom: bent models. Dashed lines: single-lined stent, dashed-dot lines: dual-lined stent. Particles were seeded uniformly throughout the aneurysm, and the residence time was computed as the time it took for the particle concentration in a 1 × 1 mm^2^ area to drop below 2% of its initial concentration. Videos 4A-F show animations of the particle washout for the six models

Residence time (RT) maps are plotted in Fig. 5. The particle washout simulations, from which RT was computed, are shown in Videos 4A-F. RT was of a similar order in the control and single-layer straight model, whereas it was strongly increased in the dual-layer straight model, with RT on both walls of the aneurysm reaching the maximal value of 20 s. In the bent models, a single area with increased RT was present in the single-lined model, with multiple zones of high RT in the dual-lined model. Maximal values in both models were on the order of 15 s, relative to 3 s in the control model.

**Fig. 6:**
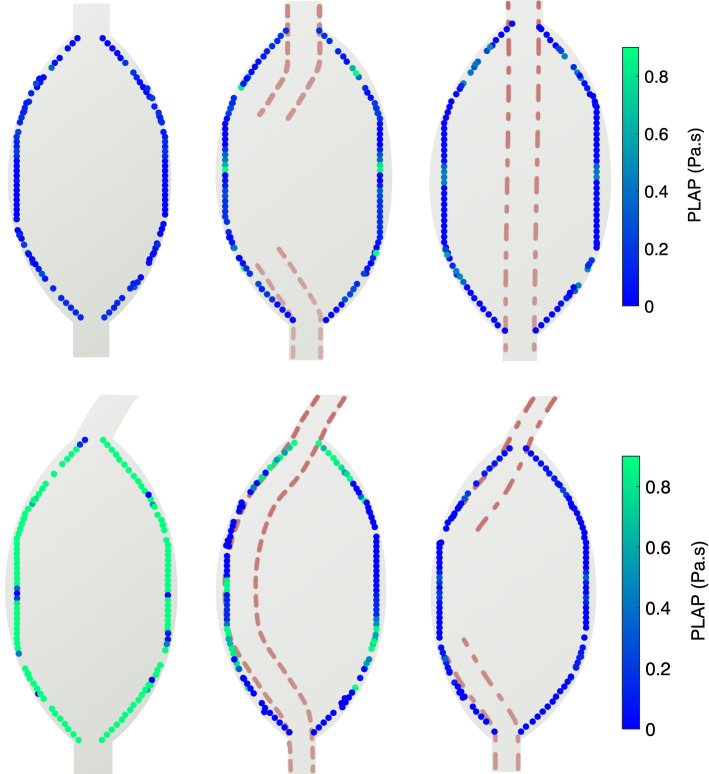
The platelet activation potential (PLAP) was computed for platelets seeded near the aneurysm wall. Top: straight models, bottom: bent models. Dashed lines: single-lined stent, dashed-dot lines: dual-lined stent. Particles were seeded along the wall at four instances in the cardiac cycle and tracked backward through time during 20 cycles to assess their shear-exposure

The platelet activation potential is shown in Fig. 6. In all models, PLAP values were substantially lower than the activation threshold of 20 Pa.s documented by lab experiments [14]. Maximal PLAP values of 0.8 Pa.s were present for the bent control model, where particles accumulated shear stress as they circulated in the large-scale vortex. The jets emanating from the stent struts in the stented models did not cause meaningfully elevated levels of shear stress.

## Discussion

This in-vitro study demonstrated that the Supera stent is capable of providing a two-to-five-fold reduction in blood flow velocities in PAAs, as well as the increasing residence time of blood particles from a few seconds to well over 10 s. These changes were meaningfully higher for a dual-lined than for a single-lined Supera stent. Especially forward flow during systole was effectively diverted, promoting flow stasis in the aneurysm, as evidenced by the prolonged residence times. Backward flow, in contrast, was less successfully diverted, highlighting the relevance of triphasic flow for flow diverting treatment of PAAs. Nonetheless, the results support the efficacy of a dual-lined Supera stent as a flow diverting configuration in PAAs.

The pulsatile flow in the popliteal artery generated distinct flow features in the straight and bent models. In the straight control model, the forward's jet momentum was too high to be overcome by backward flow, redirecting the backward flow towards the aneurysm wall. In the bent control model, forward and backward flow both acted to reinforce the counter-rotating vortex that spanned the full aneurysm. This strong aneurysm-scale vortex, which was sustained during the full cardiac cycle, led to organized flow with high velocities, which is likely to prevent intraluminal thrombus formation [15].

The introduction of a Supera stent reduced flow significantly in both straight and bent anatomies. The large reduction in the bent model could be attributed to the breakdown of the large-scale vortex, as also observed in previous analyses. The insertion of a second Supera stent in the straight model straightened the stent trajectory, optimally confining the flow within the stent pathway and reducing velocity in the aneurysm by an additional 38%. With a 27%-decrease in velocity, the Supera-in-Supera stent was not as effective in the bent model. Nevertheless, the substantial decrease in velocity supports the use of a dual Supera stent over the use of a single Supera stent, irrespective of whether the stent trajectory is changed by the second stent.

Backflow was to a significant extent not diverted through the stents, but instead through the full aneurysm. This highlights an important hemodynamic difference between cerebral and PAAs, as cerebral aneurysms are only exposed to forward flow. In the bent model, where the single and dual-lined stent had a similar trajectory, it can be appreciated that the dual-lined stent limited the expulsion of backflow relative to the single-lined stent. This further supports the packing of Supera stents to decrease total stent porosity, similar to developments for cerebral aneurysms [16]. Based on optical transmission image analysis, the Supera stent in our models had a metal area coverage in the range of 15–16% for the single-lined model and 17–23% for the dual-lined model, as compared to intracranial flow diverter designs with a 30–35% metal coverage [16]. Increasing metal coverage by additional stent layers can further reduce velocities in the aneurysm, but, with an increasingly stiff structure, the risk of stent fracture may become relevant. The optimal balance between stent packing density and risk of fracture warrants careful assessment in in-vivo studies.

The particle simulations performed in this study suggest that flow stasis is the primary factor that initiates thrombosis in PAAs treated with this flow diverting Supera stent. Particle RT was increased by a factor of 5 or more relative to the control models, with increased RT close to the aneurysm wall or the stent strut. In contrast, PLAP was not increased in the stented models relative to control models, and in general, it remained far below the threshold value for platelet activation reported in experiments [14]. For both the single and dual-lined Supera stent, the simulated platelets received elevated shear exposure when pushed through the stent struts. This, however, did not reach the level of pathological shear rates and led to a comparatively lower accumulated shear exposure than in the bent control model. The predominant importance of flow stasis suggests that (dual) antiplatelet therapy following stent placement does not necessarily inhibit aneurysm thrombosis, as confirmed in clinical pilot studies on the Supera stent [5, 6].

Two pilot studies investigated the off-label use of the Supera stent as a flow diverting configuration in patients. The first study [5] used a dual-lined Supera stent in 28 out of 31 cases and reported aneurysm thrombosis in all of them at 4-month follow-up, whereas the second [6] used a dual-lined Supera stent in 10 out of 28 cases with an aneurysm thrombosis rate of 34.5% at 12-months, both under dual-antiplatelet therapy for the first postoperative months. Both studies deployed the Supera stent with maximal compression, which maximizes the flow modulating effect and additionally benefits stent patency [17]. Whether the incidence of aneurysm thrombosis in the dual-lined vs single-lined group was higher in the second study was not reported. Although inconclusive, these results suggest that the improved hemodynamics of a dual-lined Supera stent observed in our in-vitro measurements are relevant in-vivo and may lead to improved aneurysm thrombosis rates. However, other factors, including a higher incidence of backflow, may also contribute to the difference in aneurysm thrombosis.

The two studies reported an 85% and 100% 1-year primary patency of the Supera stent, on par with an 84% primary patency of the Viabahn stentgraft [2], but no direct comparison can be made due to different inclusion criteria for aneurysm size and the number of run-off vessels. In the absence of direct comparison studies between the two approaches, the advantages and disadvantages of the respective approaches are mostly theoretical. The Viabahn stentgraft provides immediate and verifiable aneurysm exclusion with known long-term effects (10-year secondary patency 60%), with a stent fracture in 28% of cases, although this was not causally linked to loss of patency. The Supera stent’s effects on aneurysm exclusion are less rapid and more nuanced and its sizing limits it to 5–7.5 mm landing zones. The Viabahn stentgraft is likely a durable option for the majority of patients; the Supera stent approach may be considered for patients with major side branches in the aneurysm or landing zone, for tortuous PAA’s [18], for patients for whom knee flexion limitation is impractical, and for those with a contra-indication for dual-antiplatelet therapy, as no graft material is implanted. These advantages are hypothetical, however, and should be investigated in comparative clinical studies.

Several limitations deserve consideration. First, only fusiform and axisymmetric aneurysms were studied. Flows in more realistic geometries will feature more complicated three-dimensional velocities. Nonetheless, the demonstrated value of the Supera stent configuration for inducing flow stasis is unlikely to change in patient-specific geometries. Second, velocities outside the plane of interest were not measured, potentially underestimating flow velocities in the center of the aneurysm for the straight single-lined model. Nonetheless, the models where the stent was captured fully in-plane revealed that the most relevant flow phenomena occurred at the stent entrance and exit of the aneurysm, which were captured for all models.

Third, stents were deployed in a rigid, and therefore, static phantom. In reality, the popliteal artery is subject to motion which could reform the stent configuration and thereby alter the hemodynamics. We hypothesize that after aneurysm thrombosis the stent will be fixated in its configuration. This would render this effect in most cases only relevant during the first days after stenting, as aneurysm thrombosis was observed within a week for 88% of implanted Supera stents [5]. Finally, the residence time and platelet shear exposure computations used in the study provided a useful but nevertheless limited view of the complex process of thrombus initiation and propagation.

In conclusion, this study demonstrated the mechanism by which a compacted Supera stent reduces blood flow velocities and increases residence time in PAA phantoms. A dual Supera stent configuration demonstrated superior performance in these metrics relative to a single Supera stent configuration.

## Supplementary Information

Below is the link to the electronic supplementary material.

Supplementary file1 (DOCX 73 kb)

Supplementary file2 (MP4 2048 kb)

Supplementary file3 (MP4 4491 kb)

Supplementary file4 (MP4 11974 kb)

Supplementary file5 (MP4 12005 kb)

Supplementary file6 (MP4 18634 kb)

Supplementary file7 (MP4 15435 kb)

Supplementary file8 (MP4 19221 kb)

Supplementary file9 (MP4 18969 kb)

Supplementary file10 (MP4 17854 kb)

Supplementary file11 (MP4 20182 kb)

## Abbreviations

PAA: Popliteal aneurysm
WSS: Wall shear stress
PLAP: Platelet activation potential
RT: Residence time
