# Supplementary material for: The Supera Interwoven Nitinol Stent as a Flow Diverting Device in Popliteal Aneurysms
Source: Cardiovasc Intervent Radiol. 2022 Apr 4;45(6):858–66. doi: 10.1007/s00270-022-03118-x (PMC9117362; doi:10.1007/s00270-022-03118-x)
Supplement: Supplementary file 1 — Supplementary file1 (DOCX 73 kb) [file 270_2022_3118_MOESM1_ESM.docx]

**SUPPLEMENTARY MATERIAL**

**Expanded Methods**

*Phantom and flow set-up*

The anatomy of the models was based on a cohort of 10 patients with an indication for treatment of a popliteal aneurysm, i.e. a diameter >20 mm. The mean anatomic characteristics of the popliteal aneurysm are reported in Supplemental Table S1. Transparent flow phantoms were constructed in a two-step production process. The models were printed with Acylonitrile Butadiene Styrene, smoothed with acetone and subsequently secured in a rectangular box which was filled with a deaerated silicone elastomer (PDMS Sylgard 184; Dow Corning Midland, MI, USA). After curing liquid acetone was pumped through the phantoms to clear the ABS material and hence create a flow lumen in the silicone.

Duplex ultrasound measurements from the same patient group in a popliteal artery segment proximal to the aneurysm were used to derive a triphasic flow profile. Measurements were selected on the cycle-to-cycle reproducibility of the ultrasound velocity signal and the signal-to-noise ratio judged by the intensity of the doppler signal, yielding six duplex measurements. Under the assumption of a fully developed flow profile, Womersley theory[1,2] was used to convert the centerline velocity to a flow rate profile over time. The time-signal of the curves were adjusted such that peak forward flow and peak backward flow coincided for all cases. Supplemental Figure S2 displays the six resulting flow rate curves. For obtaining the patient-averaged triphasic flow rate curve used in the flow set-up, the median profile of the flow rate curves was computed for a timescale spanning 0 to 1 seconds.

For creating backflow during the end systolic phase in the flow set-up, the pump closes the inlet valve and sends a trigger signal to a solenoid valve to allow reverse flow driven by the pressurized compliance chamber distal to the PA model, analogous to the reverse flow phenomenon from the femoropopliteal artery into the renal arteries.[3] The distal flow sensors were used to set the distal resistances to match the target forward-backward volume flow ratio. A blood-mimicking fluid composed of water, glycerol and sodium iodide (47.4:36.9:15.7 weight-ratio) was used to match blood viscosity and to match the silicone refractive index.[4] The compliance chamber fluid level was set such that a physiologic pulse pressure of 50 mmHg was achieved.

*Residence time and platelet simulations*

A cycle-averaged velocity field from the particle image velocimetry results was used as input for the platelet and residence time simulations. This velocity field was modified with an inward-pointing velocity of 0.5 cm/s added to the wall points[5] to exclude particles from leaving the fluid domain through the wall.

For the residence time simulation, particles were uniformly seeded throughout the model (1200 particles per mm^2^, yielding about 19.000 particles per model) and released at timepoint 0. All particles were tracked for 20 cycles or until they left the computational domain through the inlet or outlet.

For the platelet simulations, particles were seeded along a probing distance to the aneurysm wall of 5% of the radius[5] on four evenly spread timepoints of one cycle. These particles were tracked backwards in time for a maximum of 20 cycles and the platelet activation potential (PLAP) was calculated:

$$\text{PLAP}\left( \boldsymbol{x},t \right)=\sqrt{2}\mu\int_{t-20T}^{t} \left\| \boldsymbol{D}\left( \boldsymbol{x}\left( \tau\right),\tau\right) \right\|_{F}d\tau$$

Here $\mu$ is the dynamic viscosity of the blood mimicking fluid, measured equal to 4.48 mPa.s by a rotational viscometer. Shear modulus was evaluated from the Frobenius norm of the deformation rate tensor, computed by a central differencing scheme. Results for the four timepoints were averaged to calculate one PLAP value per seeding point. Integration was stopped if the particle left the domain.

**References**

1. Womersley JR. Method for the Calculation of Velocity, Rate of Flow and Viscous Drag in Arteries when the Pressure Gradient is known. J Physiol. 1955;127:553–63.

2. McGah PM, Nerva JD, Morton RP, Barbour MC, Levitt MR, Mourad PD, et al. In vitro validation of endovascular Doppler-derived flow rates in models of the cerebral circulation. Physiol Meas. 2015;36(11):2301–16.

3. Holenstein R, Ku DN. Reverse flow in the major infrarenal vessels--a capacitive phenomenon. Biorheology. 1988;25(6):835–42.

4. Yousif MY, Holdsworth DW, Poepping TL. A blood-mimicking fluid for particle image velocimetry with silicone vascular models. Exp Fluids. 2011;50(3):769–74.

5. Di Achille P, Tellides G, Figueroa CA, Humphrey JD. A haemodynamic predictor of intraluminal thrombus formation in abdominal aortic aneurysms. Proc R Soc A Math Phys Eng Sci. 2014 Dec 8;470(2172):20140163.

**Supplemental Table S1 – Popliteal aneurysm characteristics in a 10-patient cohort with an indication for treatment**

|  | Mean ± SD |
| --- | --- |
| Anterioposterior diameter - mm | 29 ± 6.2 |
| Lateral diameter - mm | 30 ± 8.1 |
| Length - mm | 54 ± 28 |
| Neck angulation - degrees | 29 ± 6.2 |

**Supplemental Figure S2**

The computed flow rate from duplex measurements in the proximal popliteal artery of six patients with a popliteal aneurysm.
